# Adjoint multi-start-based estimation of cardiac hyperelastic material parameters using shear data

**DOI:** 10.1007/s10237-016-0780-7

**Published:** 2016-03-23

**Authors:** Gabriel Balaban, Martin S. Alnæs, Joakim Sundnes, Marie E. Rognes

**Affiliations:** 1 Simula Research Laboratory, P.O. Box 134, 1325 Lysaker, Norway; 2 Department of Informatics, University of Oslo, Blindern, P.O. Box 1080, 0316 Oslo, Norway; 3 Department of Mathematics, University of Oslo, Blindern, P.O. Box 1053, 0316 Oslo, Norway

**Keywords:** Cardiac mechanics, Adjoint equation, Parameter estimation, Hyperelasticity, Multi-start optimization

## Abstract

Cardiac muscle tissue during relaxation is commonly modeled as a hyperelastic material with strongly nonlinear and anisotropic stress response. Adapting the behavior of such a model to experimental or patient data gives rise to a parameter estimation problem which involves a significant number of parameters. Gradient-based optimization algorithms provide a way to solve such nonlinear parameter estimation problems with relatively few iterations, but require the gradient of the objective functional with respect to the model parameters. This gradient has traditionally been obtained using finite differences, the calculation of which scales linearly with the number of model parameters, and introduces a differencing error. By using an automatically derived adjoint equation, we are able to calculate this gradient more efficiently, and with minimal implementation effort. We test this adjoint framework on a least squares fitting problem involving data from simple shear tests on cardiac tissue samples. A second challenge which arises in gradient-based optimization is the dependency of the algorithm on a suitable initial guess. We show how a multi-start procedure can alleviate this dependency. Finally, we provide estimates for the material parameters of the Holzapfel and Ogden strain energy law using finite element models together with experimental shear data.

## Introduction

The personalization of computational models in cardiology is a key step toward making models useful in clinical practice and cardiac surgery. A computational model, once properly calibrated, has the potential to forecast cardiac function and disease, and can aid in planning treatments and therapies. To describe the mechanical function of the heart, the passive elasticity of the muscle tissue needs to be represented. Personalizing the effects of this elasticity in a computational model is typically accomplished by tuning a set of material parameters so that the output of the model fits observed data. Gradient-based optimization algorithms have successfully been used in the past to automatically perform the parameter tuning at an organ scale (Augenstein et al. 2005; Wang et al. 2009). In these studies, the gradient of the objective functional is approximated using one-sided finite differences.

Compared to using a global optimization method, local gradient-based methods have the advantage of using relatively few optimization iterations. This is an important consideration when optimizing organ scale finite element models, for which running a single forward model can take hours or days. On the other hand, a disadvantage of using local optimization methods is the fact that they can converge to local, globally suboptimal, minima. One way to combine the speed of a local optimization with the robustness of a global optimization is to use the multi-start method. In this method, many local optimizations are run starting from various points in parameter space and the best fitting solution of the group is taken to be the global optimum.

Another popular approach to parameter fitting is the reduced order unscented Kalman filter. This approach was successfully used to fit a transversely isotropic passive mechanics model to synthetic data (Xi et al. 2011), to partially calibrate a multi-physics model (Marchesseau et al. 2013), and to estimate regional contractility parameters (Chabiniok et al. 2012). Note however that the use of both unscented Kalman filtering and finite differences carries a computational cost that increases with the number of model parameters.

Assuming there are *k* parameters to be estimated, an unscented Kalman filter with a minimal sigma-point configuration requires $k + 1$ model evaluations at a single time level for each assimilated data point. An evaluation of a finite difference derivative on the other hand requires $k + 1$ runs of the model throughout the full span of model configurations considered.

In contrast to these two techniques, the adjoint approach computes the objective functional gradient via the solution to an adjoint equation, which involves only a single solve of a linearized system for any number of model parameters. Thus, for models involving many parameters, either due to model complexity or spatiotemporal parameter variation, the adjoint approach offers a computationally attractive approach for parameter estimation.

There are some previous results involving adjoint equations and cardiac elasticity. Sundar et al. (2009) developed a framework for the estimation of wall motion based on cine-MRI images and adjoint inversion, and Delingette et al. (2012) used an adjoint equation to estimate contractility para meters. However, both of these studies involve linear and isotropic elasticity models, which represent a significant simplification of the orthotropic and highly nonlinear behavior reported in the contemporary cardiac mechanics literature (Costa et al. 2001; Dokos et al. 2002; Holzapfel and Ogden 2009).

One reason why it is difficult to use an adjoint equation with modern nonlinear anisotropic models is the complexity required in deriving and implementing code for the solution of the adjoint problem. In order to resolve this issue, we make use of an automatic framework for generating adjoint code (Farrell et al. 2013). Here, we use this adjoint framework to estimate the material parameters of an invariant-based orthotropic myocardial strain energy law (the Holzapfel–Ogden model) (Holzapfel and Ogden 2009). This law is embedded here in an incompressible finite element framework, and we use the raw data from a simple shearing experiment (Dokos et al. 2002) as a target for optimization. These data have previously been used to estimate material parameters for a variety of other strain energy functions using a finite element framework, but with a gradient obtained using finite differences (Schmid et al. 2008, 2009). The material parameters of the particular strain energy density that we are using have also been previously estimated using digitized data based on Figure 6 of Dokos et al. (2002), and a homogeneous deformation model (Holzapfel and Ogden 2009; Wang et al. 2013; Göktepe et al. 2011). Our study is, however, the first to use the adjoint approach for the estimation of cardiac hyperelasticity parameters and the first to provide optimized material parameters for the incompressible Holzapfel–Ogden model for non-homogeneous deformations.

The rest of this paper is organized as follows. In Sect. 2 we describe the variational formulation of the elasticity model, the optimization problem for identifying the material parameters, and how the adjoint gradient formula can be used to calculate a functional gradient. In Sect. 3 we describe the verification of the forward and inverse solvers, present timings to show the efficiency of the adjoint method, and show the results of parameter estimations. Finally, we test a multi-start optimization method in order to reduce the dependence of the gradient-based algorithm on the choice of initial parameter set. We conclude by discussing our findings in Sect. 4 and drawing some conclusions in Sect. 5.

## Mathematical models and methods

We shall use the notion of the directional derivative frequently throughout. For a functional $f : Y \rightarrow \mathbb {R}$ for some vector space *Y*, we define the directional derivative of *f* with respect to the argument named $\mathbf{y}$ in the direction $\delta \mathbf{y}$

$$\begin{aligned} D_\mathbf{y} f (\mathbf{y}) [\mathbf{\delta y}] \equiv \frac{\partial }{\partial \epsilon } f(\mathbf{y} + \epsilon \ \mathbf{\delta y})\Big |_{\epsilon = 0}. \end{aligned}$$

Furthermore, we denote the total derivative by the usual notation $\frac{Df}{Dy}$ to mean the derivative of *f* with respect to all arguments depending on *y*.

### Hyperelasticity model

Let ${\varOmega }\subset \mathbb {R}^3$ be an open and bounded domain with coordinates $\mathbf{X}$ and boundary $\partial {\varOmega }$, occupied by an incompressible hyperelastic body. We consider the quasi-static regime of a body undergoing a large deformation $\mathbf{x} = \mathbf{x}(\mathbf{X})$ and are interested in finding the displacement $\mathbf{u}= \mathbf{u}(\mathbf{X}) = \mathbf{x} - \mathbf{X}$ and the hydrostatic pressure $p = p(\mathbf{X})$ that minimize the incompressible strain energy $\varPi = \varPi (\mathbf{u}, p, \mathbf{m})$:

1 $$\begin{aligned} {\varPi } (\mathbf{u}, p, \mathbf{m}) = \int _{\varOmega } \psi ( {\overline{ \mathbf C} }, \mathbf{m}) + p (J - 1) \, \mathrm {dx}\end{aligned}$$

over the space of admissible displacements and pressures satisfying any given Dirichlet boundary conditions. In (1), $\mathbf{m}$ is a set of material parameters, $J = \det \mathbf{F}$, where $\mathbf{F} = {{\mathrm{\nabla }}}\mathbf{x} = {{\mathrm{\nabla }}}\mathbf{u}+ \mathbf{I}$ denotes the deformation gradient, $\mathbf{I}$ is the identity tensor in $\mathbb {R}^3$, ${\overline{ \mathbf C} }= J^{- \frac{2}{3}} \mathbf{F}^{T} \mathbf{F}$ denotes a volume-preserving right Cauchy–Green strain tensor, and $\psi$ denotes an isochoric strain energy density.

The incompressible Holzapfel and Ogden hyperelasticity model (Holzapfel and Ogden 2009) describes large deformations and stresses in cardiac tissue via the following energy density $\psi$:

2 $$\begin{aligned} \psi (\overline{\mathbf{C}}, \mathbf{m})= & {} \, \frac{a}{2 b} \left( \exp \left[ b (I_1( \overline{\mathbf{C}}) - 3) \right] -1 \right) \nonumber \\&+ \sum _{i = f,s }\frac{h(I_{4i}(\overline{\mathbf{C}})) a_i}{2 b_i} \left( \exp \left[ b_i (I_{4i}(\overline{\mathbf{C}}) - 1)^2 \right] - 1 \right) \nonumber \\&+ \frac{a_{fs}}{2 b_{fs}} \left( \exp \left[ b_{fs} I^2_{8fs}(\overline{\mathbf{C}}) \right] - 1 \right) . \end{aligned}$$

Here *f*, *s* denote fiber and sheet directions, respectively; *h*(*x*) is a Heaviside function with a jump at $x = 1$, and the material parameters are

3 $$\begin{aligned} \mathbf{m}= (a, b, a_f, b_f, a_s, b_s, a_{fs}, b_{fs}). \end{aligned}$$

Moreover, $I_1, I_{4s}, I_{4f}, I_{8fs}^2$ are rotation invariant functions given by

4 $$\begin{aligned}&I_1({\overline{ \mathbf C} }) = {{\mathrm{tr}}}{\overline{ \mathbf C} }\nonumber \\&I_{4i}({\overline{ \mathbf C} }) = \mathbf{e}_i \cdot {\overline{ \mathbf C} }\mathbf{e}_i \quad i = f,s \nonumber \\&I_{8fs}({\overline{ \mathbf C} }) = \mathbf{e}_s \cdot {\overline{ \mathbf C} }\mathbf{e}_f \end{aligned}$$

where ${{\mathrm{tr}}}$ denotes the tensor trace and $\mathbf{e}_f, \mathbf{e}_s$ denote unit vectors pointing in the local myocardial fiber and sheet directions (Holzapfel and Ogden 2009). The strain energy density $\psi$ is rotation invariant, and polyconvex if $\mathbf{m}> {\mathbf {0}}$ (Holzapfel and Ogden 2009).

The Euler–Lagrange equations for the minimizing displacement $\mathbf{u}$ and pressure *p* of (1) read: for given $\mathbf{m}$, find $\mathbf{w}= (\mathbf{u}, p)$ such that

5 $$\begin{aligned} R(\mathbf{w}, \mathbf{m}; \mathbf{\delta w}) \equiv D_{{\mathbf{u}, p}} \varPi (\mathbf{u}, p, \mathbf{m}){[\mathbf{\delta u}, \delta p]} = 0, \end{aligned}$$

for all admissible virtual variations $\mathbf{\delta w}= (\mathbf{\delta u}, \delta p)$. Inserting the total potential energy from (1) and taking the directional derivatives, we obtain

6 $$\begin{aligned}&D_{{\mathbf{u}, p}} \varPi (\mathbf{u}, p, \mathbf{m}){[\mathbf{\delta u}, \delta p]}\\&\quad =\int _{\varOmega } \left( \left( \frac{\partial \psi ({\overline{ \mathbf C} }, \mathbf{m})}{\partial \mathbf{F}} + {p} J \mathbf{F}^{-T} \right) : {{\mathrm{\nabla }}}\mathbf{\delta u}+ (J - 1) \delta p\right) \, \mathrm {dx}.\nonumber \end{aligned}$$

### Parameter estimation as a PDE-constrained optimization problem

In the general case, the passive material parameters $\mathbf{m}$ entering the constitutive relationship (2) are not known. In order to estimate these parameters from data, we propose to use a numerical approximation in combination with a gradient-based optimization algorithm in which the gradients are computed via an adjoint model. The optimization algorithm seeks to minimize the misfit between model output and observations. Denoting the misfit functional by $I = I(\mathbf{w}(\mathbf{m}), \mathbf{m})$, the optimization problem reads:

7 $$\begin{aligned}&\min _\mathbf{m}I(\mathbf{w}(\mathbf{m}), \mathbf{m}) \quad \text {subject to} \quad R(\mathbf{w}, \mathbf{m}; \mathbf{\delta w}) = 0 \quad \forall \mathbf{\delta w}\in W,\nonumber \\ \end{aligned}$$

together with suitable Dirichlet boundary conditions on $\mathbf{w}$. We also require that $\mathbf{m}> 0$ to ensure the functional (1) is polyconvex (Holzapfel and Ogden 2009). For notational convenience, we will sometimes use the reduced formulation of the misfit functional and its gradient with respect to the material parameters $\mathbf{m}$. In particular, we introduce the reduced functional $\hat{I}$

8 $$\begin{aligned} \hat{I}(\mathbf{m}) \equiv I(\mathbf{w}(\mathbf{m}), \mathbf{m}). \end{aligned}$$

In our numerical experiments, we use Sequential Least Squares Programming (SLSQP) as implemented in Kraft (1988) and wrapped in the package SciPy (Jones et al. 2001) in order to solve (7).

### Multi-start Optimization

A common challenge with gradient-based algorithms is that the solution obtained depends on the choice of initialization point for the algorithm. Moreover, the optimized solution may be a local minimum only and not necessarily a global minimum. One way to attack these issues is to run many optimizations from randomly chosen initial parameter points and to chose the resulting optimized material parameter set that gives the best fit. This method is often referred to as multi-start optimization (Boender and Kan 1987) and is an example of combining global and local optimization.

Due to the presence of exponential functions in the strain energy (2), it is possible for calculated stresses to become very large, which may result in convergence issues for the numerical solution of the Euler–Lagrange equation (5). This can easily occur if several material parameters have large values. In order to minimize this problem, we have designed a procedure to generate random initial guesses which limits the number of large material parameter values while still allowing for a large range of initial possible values for each parameter. The procedure works as follows: first set a maximum parameter value $P_\mathrm{max}$. Then choose *N* (with $N = 8$ in our case) points $p_i, \ i \in \{1,2,3\ldots n \}$, from a uniform distribution defined over the interval $[0, P_\mathrm{max}]$ and let $p_0 = 0$. The parameter values $m_i$ are then set to be the distances between successive randomly drawn points, that is $m_i = p_i - p_{i - 1}$.

### Computing the functional gradient via the adjoint solution

Gradient-based optimization algorithms in general, and the SLSQP algorithm in particular, rely on the total derivative of the objective functional (8). By introducing an *adjoint* state variable, this derivative may be computed efficiently. We summarize this result below. Our presentation is based on Gunzburger (2003), and is adapted here to the solid mechanics setting.

We define three abstract spaces *W*, *M*, and ${\varPhi }$, where *W* is the space of all possible solutions to the variational equation (5) which also satisfy any given Dirichlet boundary conditions, *M* is the material parameter vector space, and ${\varPhi }$ is the space of virtual variations. The Lagrangian $L: W \times M \times {\varPhi }\rightarrow \mathbb {R}$ is defined as:

9 $$\begin{aligned} L(\mathbf{w}, \mathbf{m}, {\mathbf \phi }) = I(\mathbf{w}, \mathbf{m}) - R(\mathbf{w}, \mathbf{m}; {\mathbf \phi }). \end{aligned}$$

For all $\mathbf{m}\in M$, $\mathbf{w}\in W$ solving the state equation (5), we have

$$\begin{aligned} \frac{D}{D \mathbf{m}} R(\mathbf{w}(\mathbf{m}), \mathbf{m}; \phi ) = 0, \end{aligned}$$

such that the total derivatives of *I* and *L* coincide,

10 $$\begin{aligned} \frac{D}{D \mathbf{m}} I(\mathbf{w}(\mathbf{m}), \mathbf{m}) = \frac{D}{D \mathbf{m}} L(\mathbf{w}(\mathbf{m}), \mathbf{m}, {\mathbf \phi }). \end{aligned}$$

If we choose ${\mathbf \phi }\in \varPhi$ such that

11 $$\begin{aligned} D_{\mathbf{w}} L(\mathbf{w}, \mathbf{m}, {\mathbf \phi })[\delta \mathbf{w}] = 0 \end{aligned}$$

for all $\delta \mathbf{w}\in W$, which in particular includes $\delta \mathbf{w}= D_{\mathbf{m}} \mathbf{w}(\mathbf{m}){[\mathbf{\delta m}]}$, the total derivative of *L* with respect to $\mathbf{m}$ in the direction $\mathbf{\delta m}$ simplifies as follows using the chain rule:

12 $$\begin{aligned} \frac{D}{D \mathbf{m}} L(\mathbf{w}(\mathbf{m}), \mathbf{m}, {\mathbf \phi })= & {} D_{\mathbf{w}} L(\mathbf{w}, \mathbf{m}, {\mathbf \phi }) [ D_{ \mathbf{m}} \mathbf{w}(\mathbf{m}){[\mathbf{\delta m}]} ]\nonumber \\&+ D_{\mathbf{m}} L(\mathbf{w}, \mathbf{m}, {\mathbf \phi }){[\mathbf{\delta m}]}\nonumber \\= & {} D_{\mathbf{m}} L(\mathbf{w}, \mathbf{m}, {\mathbf \phi }){[\mathbf{\delta m}]} \end{aligned}$$

Then, for any infinitesimal variation in the material parameters $\mathbf{\delta m}$, combining (10), (12), and (9) yields an efficient evaluation formula, not requiring derivatives of the state variable $\mathbf{w}$ with respect to the material parameters $\mathbf{m}$, for the total derivative of *I*:

13 $$\begin{aligned} \frac{D}{D \mathbf{m}} I(\mathbf{w}(\mathbf{m}), \mathbf{m})= & {} D_{\mathbf{m}} I(\mathbf{w}, \mathbf{m}){[\mathbf{\delta m}]}\nonumber \\&- D_{\mathbf{m}} R(\mathbf{w}, \mathbf{m}, {\mathbf \phi }){[\mathbf{\delta m}]}. \end{aligned}$$

We still need to compute ${\mathbf \phi }$. By defining the form $R_{\mathbf{w}}$ and its adjoint $R_{\mathbf{w}}^*$,

$$\begin{aligned} R_{\mathbf{w}} (\mathbf{w}, \mathbf{m}; \delta \mathbf{w}, {\mathbf \phi })\equiv & {} D_{\mathbf{w}} R(\mathbf{w}, \mathbf{m}; {\mathbf \phi }){[\delta \mathbf{w}]}, \\ R_{\mathbf{w}}^{*} (\mathbf{w}, \mathbf{m}; {\mathbf \phi }, \delta \mathbf{w})\equiv & {} R_{\mathbf{w}} (\mathbf{w}, \mathbf{m}; \delta \mathbf{w}){[{\mathbf \phi }]}, \end{aligned}$$

we can rewrite (11) as

$$\begin{aligned} D_{\mathbf{w}} L(\mathbf{w}, \mathbf{m}, {\mathbf \phi }) [\delta \mathbf{w}]= & {} D_{\mathbf{w}} I(\mathbf{w}, \mathbf{m})[\delta \mathbf{w}]\\&- R_{\mathbf{w}}^{*} (\mathbf{w}, \mathbf{m}; {\mathbf \phi }, \delta \mathbf{w})= 0, \end{aligned}$$

and thus recognize the adjoint equation: given $\mathbf{m}$, $\mathbf{w}$, find ${\mathbf \phi }\in \varPhi$ such that

14 $$\begin{aligned} R_{\mathbf{w}}^{*} (\mathbf{w}, \mathbf{m}; {\mathbf \phi }, \delta \mathbf{w}) = D_{\mathbf{w}} I(\mathbf{w}, \mathbf{m})[\delta \mathbf{w}] \end{aligned}$$

for all $$\delta \mathbf{w}\in W$$.

In summary, the adjoint-based gradient evaluation formula is: given $\mathbf{m}$, first compute $\mathbf{w}$ by solving the state equation (5), next compute ${\mathbf \phi }$ by solving (14), and finally evaluate (13).

### Description of shearing experiments

We aim to optimize the material parameters of the Holzapfel–Ogden model (2) with respect to target experimental data, in particular data resulting from an earlier set of simple shearing experiments (Dokos et al. 2002). In these experiments, 6 pig hearts were extracted. From each heart, three adjacent $3 \text {mm} \times 3 \text {mm} \times 3 \text {mm}$ cubic blocks were cut in such a way that the sides of the cubes were aligned with the local myocardial fiber and sheet directions. A device held two opposing faces of each cube between two plates using an adhesive. The top plate was displaced in order to put each specimen in simple shear. For each specimen, 6 different modes of shear were tested. These modes are described using the *F*, *S*, *N* coordinate system, which refer to the myocardial fiber, sheet and sheet normal directions, respectively. Each mode is denoted by two letters, where the first defines the normal of the face of the cube that is being displaced, and the second refers to the direction of displacement. These 6 modes are $\textit{FS}, \textit{FN}, \textit{SF}, \textit{SN}, \textit{NF}, \textit{NS}$.

In order to remove the effects of strain softening, preliminary displacements were applied to the tissue samples until no further softening was observed. After that, displacements were once again applied, and the forces in the shear direction were measured on the top plate. These measurements were taken for circa 200–250 various states of shear per mode.

**Fig. 1:**
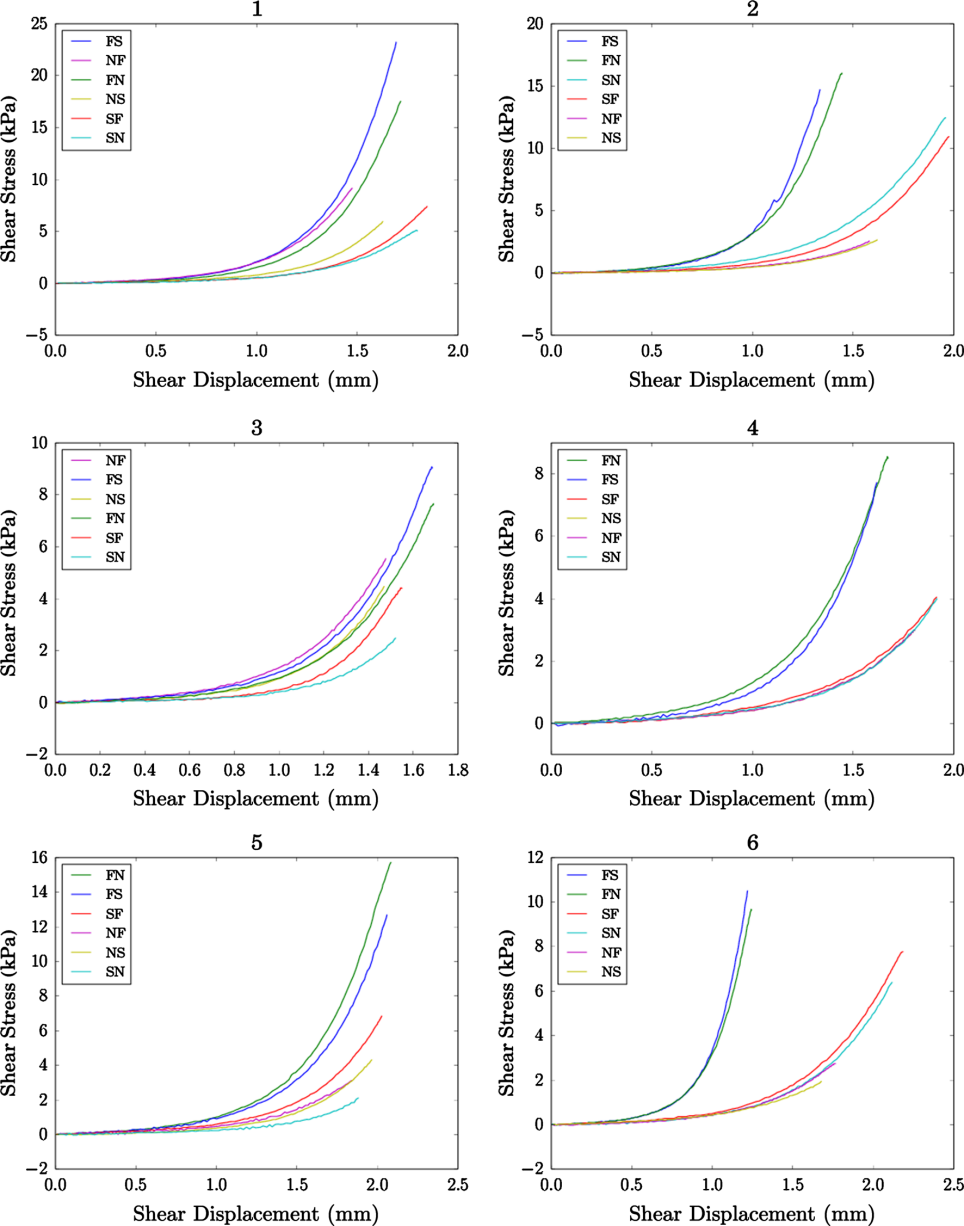
Stress–strain relations, numbered 1 through 6, obtained from simple shearing experiments performed on $3\,\text {mm} \times 3\,\text {mm} \times 3\,\text {mm}$ cubes of myocardium extracted from 6 porcine hearts. The modes are ordered from highest to lowest stiffness in each experiment. The data originate from the study Dokos et al. (2002), but were not published in the subsequent article. In Experiment 4 the data for one of the NS–NF *curves* were copied into the other before we received it, so the two *curves* lie here on top of one another

In Fig. 1 we display the stress–strain relations for positive displacements that were obtained from the shearing experiments (Dokos et al. 2002). As can be seen in Figures 4 and 6 of Dokos et al. (2002), the experimentally obtained curves contain a high degree of symmetry through the line $y = -x$. We can expect the same symmetry in the stresses computed by finite element models which use the strain energy (2) since changing the sign of the displacement map will change the sign of the resulting stresses but preserve their magnitude. In the previous studies Holzapfel and Ogden (2009), Göktepe et al. (2011), and Wang et al. (2013), only the data for positive shear displacements were used. For the sake of comparability, we restrict our data in the same way.

In our numerical experiments, we use two data sets with reference to the numbering of Dokos et al. (2002). The first is Data Set 6, and the second data is Data Set 2 with the *SF* and *SN* curves swapped. This swap and the choice of data sets are discussed further in Sect. 4. For clarity, we shall refer to Data Set 6 as “transversely isotropic” and Data Set 2 with the swap as “orthotropic”, as the respective stress–strain curves are typical of materials of these types. For each mode, the prescribed shear displacement is modeled as a Dirichlet boundary condition for the displacement on the respective top and bottom faces in the respective direction.

### Choice of objective functional

In order to estimate the passive material parameters of the Holzapfel–Ogden model, we make use of a least squares objective functional. This functional defines a distance from the model output to the data points of the shearing experiment, and we seek the material parameter set $\mathbf{m}$ that minimizes this. Before introducing our objective functional, we define the set of directions $\mathcal {D} = \{F, S, N\}$, referring to fiber, sheet and sheet normal directions. We also use the notation (*i*, *j*) to refer to a mode, with the index *i* referring to the normal of the face that is shifted, and *j* to the direction in which the shift occurs.

Our fit function is similar to that used in Schmid et al. (2007) and is given by

15 $$\begin{aligned} \hat{I}(\mathbf{m})^2 = \sum _{i \in \mathcal {D}} \sum _{j \in \mathcal {D}} \sum _{k=1}^G \omega _k \left( t^{i,j}_{\text {model}}({c}_k, \mathbf{m}) - t^{i,j}_{\text {exper}}({c}_k) \right) ^2 \end{aligned}$$

In (15), $t^{i,j}_{\text {exper}}$ is the force measured during the experiment, and $t^{i,j}_{\text {model}}$ is the force generated by the finite element model at each prescribed shear displacement ${c}_k \in [0, {C}^{i,j}]$, where ${C}^{i,j}$ is the maximal prescribed displacement of the mode (*i*, *j*) in the experiment. Each ${c}_k$ is chosen to be a Gauss point of a *G*-point Gauss integration rule defined over $[0, {C}^{i,j}]$, and $\omega _k$ is the value of the Gauss weight related to ${c}_k$. Explicitly, for mode (*i*, *j*) with top face $\partial {\varOmega }_i$, $t^{i,j}_{\text {model}}$ is given by

16 $$\begin{aligned} t^{i,j}_{\text {model}}({c}_k, \mathbf{m}) = \int _{\partial \varOmega _{i}} \frac{\partial \psi (\mathbf{u}({c}_k), \mathbf{m})}{\partial \mathbf {F}_{i,j}} \, \text {d}S, \end{aligned}$$

where $\mathbf {F}_{i,j} = \mathbf {e}_{i} \cdot \mathbf {F} \mathbf {e}_{j}$ is a shear component of the deformation gradient.

Evaluating the inner loop of $\hat{I}$ requires solving (5) once for each given shear displacement ${c}_k$. The motion given by the calculated displacements is then a quasi-static approximation of the motion undergone by the corresponding tissue in the shearing experiment.

Following Schmid et al. (2007), we evaluate the least squares fit (15) at *G* Gauss integration points, rather than for all 250 recorded points for each shear mode, in order to greatly reduce the computational expense of evaluating $\hat{I}$. At each Gauss point, we obtain the corresponding shear stress by linearly interpolating between the two neighboring stresses which were recorded in the experiments of Dokos et al. (2002).

The use of Gauss integration is based on the observation that $\hat{I}(\mathbf{m})$ is an approximation to the following expression

17 $$\begin{aligned} {\left( \sum _{j \in \mathcal {D}} \sum _{i \in \mathcal {D}} \int _0^{C^{i,j}} \left( t^{i,j}_{\text{ model }}(c, \mathbf{m}) - t^{i,j}_{\text{ exper }}(c) \right) ^2 \ \mathrm{d}c \right) ^{\frac{1}{2}}.} \end{aligned}$$

By setting $t^{i,j}_{\text{ model }} = 0$ and approximating the integral by the midpoint rule applied to the full dataset, we can determine the quality of the Gauss approximation. In order to do this, we define the relative error

18 $$\begin{aligned} {\epsilon _\mathrm{rel} = \left| \frac{ \hat{I} - \hat{I}_\mathrm{mid}}{\hat{I}_\mathrm{mid}} \right| ,} \end{aligned}$$

where $\hat{I}_\mathrm{mid}$ is the midpoint rule approximation of (17) evaluated over the full data, and $\hat{I}$, given by (15), is evaluated at a reduced set of Gauss points. We noticed that 9 Gauss points are sufficient to reduce $\epsilon _\mathrm{rel}$ to less than 0.01. However, in our numerical experiments we use $G = 40$ Gauss points as this guaranteed small enough changes in the solution of the Euler–Lagrange equation (5) from one Gauss point to the next, so that our Newton’s method solution of (5) always converged.

### Finite element discretization of the hyperelasticity equations

We represent each tissue sample of the shearing experiments by a three-dimensional cube ${\varOmega }= [0, 3]^3 (\text {mm}^3)$. An $N \times N \times N$ mesh of this cube was constructed by uniformly dividing the mesh into $N \times N \times N$ boxes and then subdividing the boxes into tetrahedra. The local myocardial fiber and sheet orientations were represented as spatially constant vectors aligned with the coordinate axes.

On these geometries, we solve (5) and its adjoint, using a Galerkin finite element method with the Taylor–Hood finite element pair (Hood and Taylor 1974); e.g., a continuous piecewise quadratic vector field for the displacement and a continuous piecewise linear scalar field for the pressure. For the solution of the nonlinear system of equations, we use a Newton trust region method. The absolute tolerance of the nonlinear solver was set to $10^{-10}$ in the numerical experiments below. Linear systems are solved by LU factorization.

**Fig. 2:**
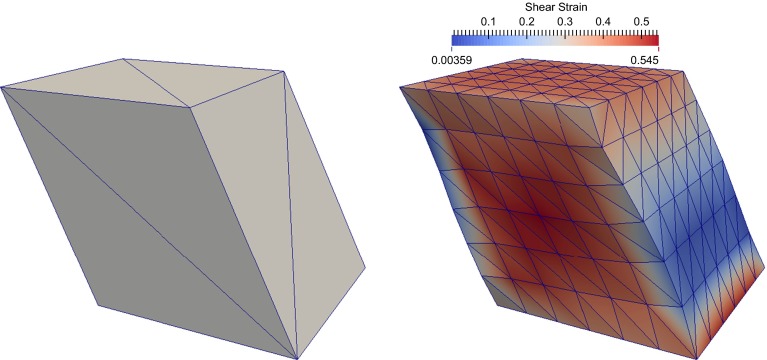
Finite element representation of cubes of cardiac tissue undergoing simple shear in the NS mode. The *bottom* of the cube is fixed, and the top displacement is given. *Left* homogeneous deformation with a constant shear angle. *Right* finite element solution on a $6 \times 6 \times 6$ mesh. The *plot* shows the value of the NS-component of the right Cauchy–Green strain tensor $\mathbf {C}$

Additionally, we model the case of a homogeneous deformation which corresponds to a linear displacement with a constant shear angle throughout the domain. Such a model can be represented by discretizing the cubes with a single layer of linear finite elements: the resulting displacement is completely determined by the prescribed boundary conditions. Figure 2 illustrates the two kinds of deformations on cube meshes.

The discrete variational formulation of the Euler–Lagrange equations is implemented using the FEniCS Project software (Alnæs et al. 2014; Logg et al. 2011) and dolfin-adjoint (Farrell et al. 2013). From a FEniCS forward model, dolfin-adjoint automatically generates the symbolic adjoint system of equations and computes the functional gradient (13) using the adjoint solution. The FEniCS framework automatically generates and compiles efficient C++ code for the assembly of the relevant linear systems from the symbolic representations of both forward and adjoint equations, and solves the nonlinear and linear systems using e.g., PETSc (Balay et al. 2015). With this setup, we observed that a typical solution of the Euler–Lagrange equation (5) takes 6 Newton iterations.

**Table 1:** Synthetic data test results

	*a*	*b*	$a_f$	$b_f$	$a_s$	$b_s$	$a_{fs}$	$b_{fs}$	*I*
	(kPa)		(kPa)		(kPa)		(kPa)		(mN)
Initial	0.059	8.023	18.472	16.026	2.481	11.120	0.216	11.436	
Target (80 %)	0.047	6.418	14.778	12.821	1.985	8.896	0.173	9.149	
Homogeneous	0.047	6.418	14.778	12.821	1.985	8.896	0.173	9.149	4.611 $\times 10^{-8}$
Finite Element	0.047	6.406	14.778	12.821	1.983	8.938	0.173	9.155	0.00082

The first row (Initial) contains the material parameter values used to initialize the algorithm, while the second row (Target) contains the parameters that were used to generate the synthetic stresses. The rows marked ’Homogeneous’ and ’Finite Element’ contain optimized parameter values coming from homogeneous deformation and finite element models. These optimized values are matched perfectly by the optimized homogeneous model and very closely by the finite element model

## Numerical results

### Verification

Each of the finite element, adjoint, and optimization solvers have been carefully verified, separately and combined, as follows:

(i) The finite element solver was verified by the method of manufactured solutions (Salari and Knupp 2000). Following this method, we chose an analytic expression for the displacement and pressure fields
  19 $$\begin{aligned} \begin{array}{l} {\mathbf{u}= \left( t x^3, \ y \left( \frac{1}{3tx^2 + 1} -1 \right) , 0 \ \right) } \\ {p = 0.} \end{array} \end{aligned}$$
  Here *x*, *y* refer to Cartesian coordinates and *t* is a scaling parameter which we set to $t = 0.2$. Using this analytic expression we derived Dirichlet boundary conditions over a unit cube, and a loading term *f* which satisfied a pointwise form of Eq. (6)
  20 $$\begin{aligned} { \frac{\partial \psi ({\overline{ \mathbf C} }, \mathbf{m})}{\partial \mathbf{F}} + p J \mathbf{F}^{-T}} {= f \quad \text{ in } \varOmega .} \end{aligned}$$
  Note that the chosen displacement field satisfies the incompressibility constraint $J -1 = 0$. We then computed finite element approximations to (19) and observed the expected second-order convergence of the displacement gradient to the analytical displacement gradient (Hood and Taylor 1974).
(ii) We verified the computation of stresses in the finite element model by prescribing a homogeneous deformation and comparing the resulting numerically integrated top face shear stress values to analytically computed values. The analytic values were based on the calculations found in Holzapfel and Ogden (2009, Section 5a), and the numerical values were observed to match closely.
(iii) We confirmed the correctness of the adjoint gradients by considering the linearization of the functional $\hat{I}(\mathbf{m})$ around $\mathbf{m}$ with perturbation $\Delta \mathbf{m}$ and using Taylor’s theorem: the expression
  21 $$\begin{aligned} \quad {\hat{I}(\mathbf{m}) - \hat{I}(\mathbf{m}+ \Delta \mathbf{m}) + \frac{D \hat{I}(\mathbf{m})}{D \mathbf{m}} (\Delta \mathbf{m}) = O\left( \Delta \mathbf{m}^2\right) } \end{aligned}$$
  converged to 0 at a rate of 2 as $\Delta \mathbf{m}\longrightarrow 0$, which can only be expected if $\frac{D \hat{I}(\mathbf{m})}{D \mathbf{m}}$ is computed accurately.

### Parameter estimation with synthetic data

Additionally, we verified the optimization solver by performing a synthetic data test. In this test we chose a target set of material parameters, Table 1, 2nd line, and used them to compute synthetic integrated stress values for all 6 shear modes of the tissue experiment (Dokos et al. 2002). These synthetic stresses were then matched by an optimization starting from material parameter values $25\,\%$ higher than the target.

We performed this test using our two models for deformation. The first model assumed a homogeneous shear angle through the material and the second model was a finite element model with a $1 \times 1 \times 1$ mesh. Since the displacement field of the finite element model was element-wise quadratic, it allowed for more flexibility in the deformation field. The results of this synthetic data test are presented in Table 1 and show that the optimization algorithm was able to closely match the target material parameters.

### Parameter estimation with experimental stress data

In the following, we present the results of fitting the Holzapfel–Ogden strain energy law (2) using the objective function (15) and a SLSQP optimizer with bound constraints. The SLSQP algorithm makes use of the gradient of the objective functional which we obtain using the adjoint gradient formula (13).

As the numerical solution of the nonlinear Euler–Lagrange equation (5) easily fails to converge when a material parameter becomes too small, we set a lower bound of $1.0 \times 10^{-2}$ on the components of $\mathbf{m}$ while optimizing finite element models. This bound was not necessary for the homogeneous deformation models as no Euler–Lagrange equation is solved. All optimizations were carried out until the optimizer was unable to further reduce the objective functional or an absolute tolerance of $1.0\times 10^{-6}$ in the 2-norm of the functional gradient was reached.

#### Material parameter estimation using *a priori* knowledge

The material parameters of the Holzapfel–Ogden model have previously been estimated using a homogeneous deformation model (Table 1, 2nd row in Holzapfel and Ogden 2009). We first used these values as the initial values for optimization of our homogeneous model targeting the transversely isotropic and orthotropic data sets. The optimized results are listed in Table 2 with the label Homogeneous.

**Fig. 3:**
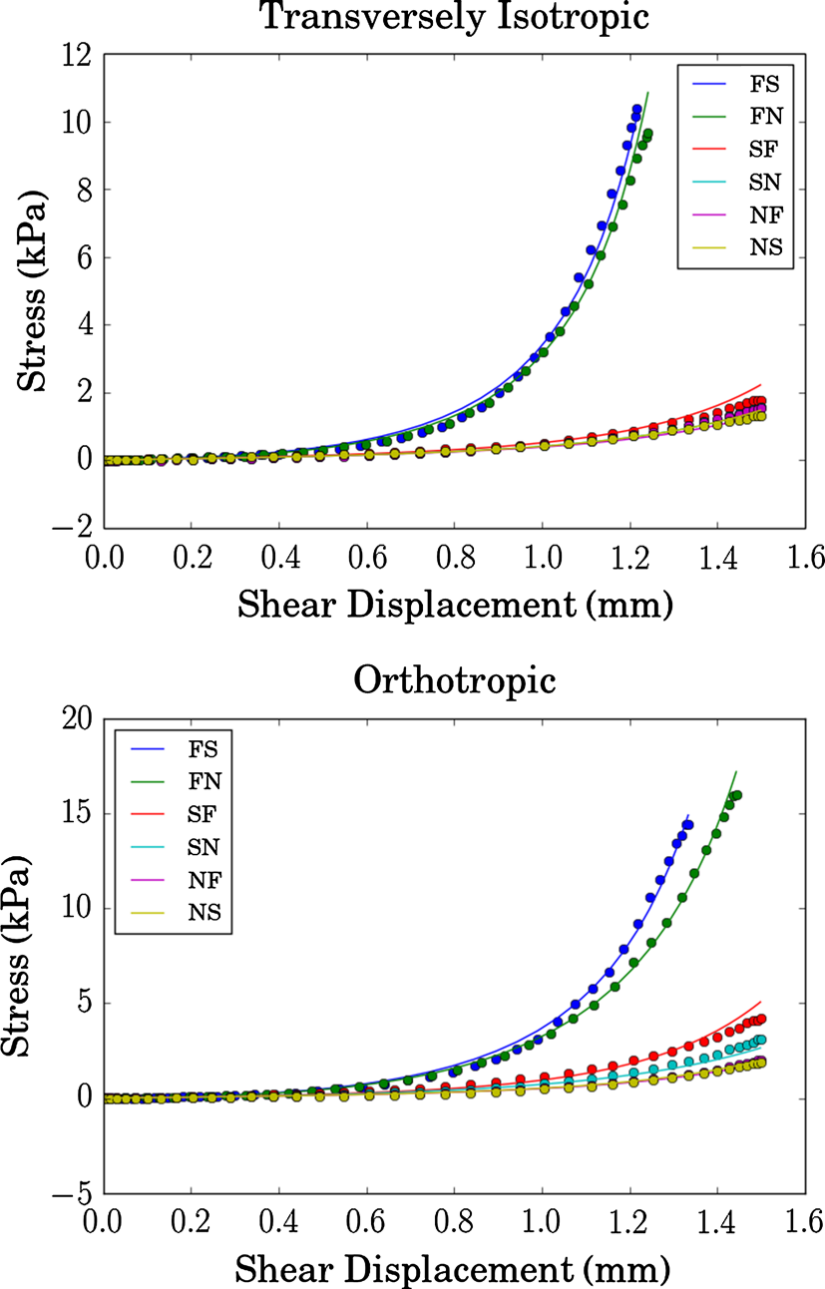
Comparison of optimized model stress–strain curves with experimental data. The *dots* are interpolated experimental data at Gauss points, the *solid lines* show the output of the finite element models with $N = 8$ elements per edge of the cube

We next consider finite element models that allow for heterogeneous shear displacements. Beginning with a $1 \times 1 \times 1$ cube and the optimal material parameters from the homogeneous model as initial values, we computed optimal values for the $1 \times 1 \times 1$ case. This procedure was repeated for $N \times N \times N$ cubes with $N = 2, 4, 6, 8$, using the results of the previous optimization as the initial condition for the next case. The resulting parameter values are presented in Table 2, and the corresponding optimal stress–strain curves are shown in Fig. 3.

We note that going from $N = 8$ to $N = 10$ using both the transversely isotropic and the orthotropic data does not change the material parameters rounded to two 2 significant digits, and therefore consider our finite element models to be sufficiently refined at this resolution. We also note that the fit values, *I*, decreased with mesh refinement up to about 2 digits accuracy. We expect this decrease since increased mesh refinement gives more flexibility in the deformation field of the finite element model.

**Table 2:** Material parameters fitted to the orthotropic and transversely isotropic datasets for the Homogeneous and $N \times N \times N$ finite element models

	*a*	*b*	$a_f$	$b_f$	$a_s$	$b_s$	$a_{fs}$	$b_{fs}$	*I*	Ev.	Grad
	(kPa)		(kPa)		(kPa)		(kPa)		(mN)		Ev.
*Transversely isotropic*											
Homogeneous	0.544	6.869	23.220	39.029	0.0001	0.172	0.248	5.310	3.291	41	21
N = 1	0.593	6.841	23.209	38.826	0.010	0.010	0.243	9.531	3.173	44	37
N = 2	0.732	6.818	22.110	39.946	0.010	0.010	0.183	13.614	3.010	24	18
N = 4	0.807	6.737	21.349	40.468	0.010	0.010	0.122	17.936	2.819	25	18
N = 6	0.794	6.859	21.212	40.537	0.010	0.010	0.129	17.462	2.802	22	15
N = 8	0.784	6.973	21.149	40.584	0.010	0.010	0.145	16.401	2.815	21	14
N = 10	0.778	7.048	21.112	40.585	0.010	0.010	0.150	16.036	2.819	24	17
*Orthotropic*											
Homogeneous	0.556	7.940	33.366	14.224	2.804	0.0001	0.588	8.216	6.804	31	20
N = 1	0.766	6.857	31.640	15.210	2.069	0.010	0.352	15.243	5.880	29	19
N = 2	1.040	6.557	29.375	15.979	1.742	0.010	0.118	23.296	4.565	39	24
N = 4	0.979	7.364	28.882	15.813	2.058	0.010	0.107	24.039	3.952	28	16
N = 6	0.961	7.495	28.762	15.783	2.088	0.010	0.114	23.549	3.899	21	13
N = 8	0.962	7.510	28.649	15.806	2.044	0.010	0.122	23.027	3.899	20	11
N = 10	0.959	7.542	28.565	15.813	2.017	0.010	0.123	22.750	3.981	25	12

*I* refers to the value of the objective functional. The number of functional evaluations (Ev.) and functional gradient evaluations (Grad Ev.) are given in the two rightmost columns

#### Material parameter estimation using multi-start optimization

In this section, we present the results of using the multi-start method to estimate the optimal material parameters, rather than relying on a good initial guess. For the calculation of random initial guesses, we set $P_\mathrm{max} = 40$, cf. Sect. 2.3. This value is close to the largest material parameter found in Table 2. Note that this choice gives a conservative set of initial parameters for the optimization algorithm (low initial values) which in turn enhances the robustness of the procedure. We also set 60 as an upper bound for each material parameter value during the optimization. Without this upper bound, we observed that many optimizations crashed or converged to suboptimal local minima.

In each multi-start experiment, 30 random starting points were used. The mesh fineness was set to the level of $N = 8$, which was sufficient to give converged material parameter sets when using a priori knowledge in Sect. 3.3.1. In Table 3 we present the best fitting results of the multi-start experiments and note that they are very close to those obtained with a priori knowledge in Table 2.

**Table 3:** Results of fitting material parameters to the transversely isotropic and orthotropic data sets using the multi-start method

	*a*	*b*	$a_f$	$b_f$	$a_s$	$b_s$	$a_{fs}$	$b_{fs}$	I
	(kPa)		(kPa)		(kPa)		(kPa)		(mN)
*Transversely isotropic*									
*N* = 8	0.784	6.973	21.149	40.584	0.010	0.010	0.145	16.401	2.815
Multistart Best Fit	0.795	6.855	21.207	40.545	0.010	0.010	0.130	17.446	2.802
*Orthotropic*									
*N* = 8	0.962	7.510	28.649	15.806	2.044	0.010	0.122	23.027	3.899
Multistart Best Fit	0.964	7.510	28.654	15.791	2.051	0.010	0.118	23.230	3.959

The rows labeled ’Multistart Best Fit’ correspond to the optimizations with the lowest misfit value *I*. The rows labeled ’$N= 8$’ are copied from Table 2 for reference

#### Objective functional values for alternative material parameters

Several other studies Holzapfel and Ogden (2009), Göktepe et al. (2011), Wang et al. (2013) have used the Dokos et al. (2002) shear data to calibrate the Holzapfel and Ogden strain energy (2). These studies used homogenized deformation models for the optimization. In Table 4 we list the computed objective functional value of parameter sets originating from previous studies using the orthotropic dataset and finite element model $(N = 8)$. The results indicate that our parameter set fits these data better than the previously computed ones.

We also note that our finite element parameter set with finite element model has a better fit value than the homogeneous parameter set with the homogeneous model. Indeed, we expect the finite element fit to be at least as good as the homogeneous fit, as the finite element model allows for greater flexibility in the deformation field, above and beyond that of the homogeneous model.

### Computational efficiency of the adjoint-based functional gradient

Adjoint solver efficiency may be measured by comparing the runtime of the adjoint and forward solves. Here, we examine the overall gradient efficiency in a similar manner. We consider the evaluation of the gradient of the objective functional (15), though in a reduced case with only a single shear mode included in the sum and a reduced forward solve consisting of a single nonlinear solver iteration. In this case, the forward and adjoint models each consist of a single linear solve in addition to a number of residual evaluations. For larger linear system sizes, the runtime of a linear solve is expected to dominate the runtime of assembly, and thus these forward and adjoint models are of roughly the same computational expense.

**Fig. 4:**
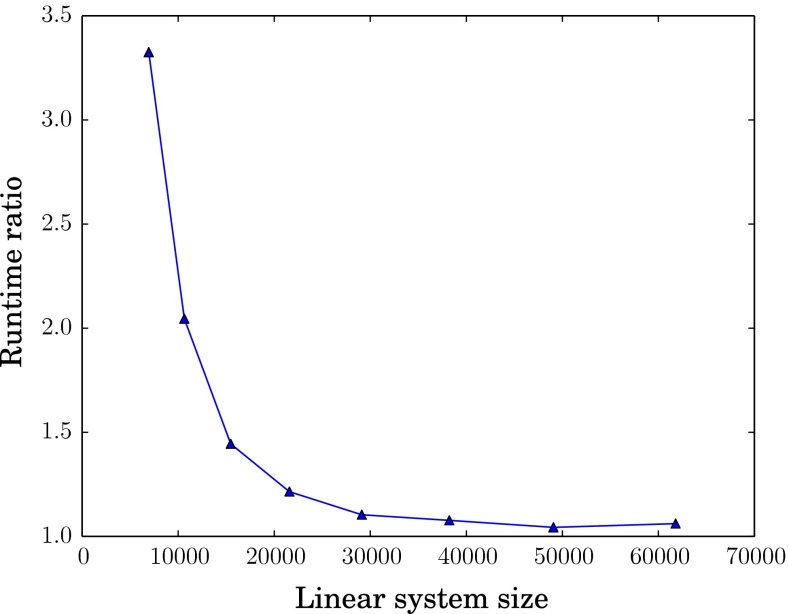
Gradient efficiency: ratio of gradient evaluation runtime over single Newton iteration runtime for increasing linear system sizes

For this reduced case, we evaluated the adjoint-based gradient for a range of linear system sizes. For each system size, we calculated the gradient runtime ratio; that is, the runtime used by the evaluation of the gradient divided by the runtime of the forward solve. The resulting ratios are plotted in Figure 4. The curve indicates that the gradient run-time ratio gets close to the theoretically optimal value of 1 as we increase the system size.

## Discussion

### Choice of shearing experiment datasets

Of the six shearing experiment datasets, cf. Figure 1, we have used two for parameter estimation. One of the reasons for this choice is an incompatibility of most of the datasets with assumptions made in the design of the strain energy functional (2). In particular, the strain energy (2) dictates an ordering of the shear mode stiffnesses in the case of a homogeneous shear displacement. We can see this by adapting the analysis that leads to equations (5.23)–(5.28) of Holzapfel and Ogden (2009). In this analysis, a parameter $\gamma$ is introduced to represent the amount of simple shear displacement present in a homogeneous deformation. For example for the FS mode

22 $$\begin{aligned} {\mathbf{F} = \begin{bmatrix} 1&\quad \gamma&\quad 0 \\ 0&\quad 1&\quad 0 \\ 0&\quad 0&\quad 1 \end{bmatrix}.} \end{aligned}$$

Using this deformation gradient, and the respective deformation gradients of the other modes, the shear component of the Cauchy stress $\sigma$ in the shearing direction can be calculated for each mode. If we consider the same invariants as in (2), that is $I_1, I_{4f}, I_{4s}, I_{8fs}$, and use the notation $\psi _i = \frac{\partial \psi }{\partial I_{i}}$, we arrive at the following equations for shear stress as a function of shear displacement

23 $$\begin{aligned} {\text{(FS): } \quad \sigma _{FS}}= & {} 2(\psi _1 + \psi _{4f})\gamma + \psi _{8fs}, \nonumber \\ {\text{(FN): } \quad \sigma _{FN}}= & {} 2(\psi _1 + \psi _{4f})\gamma , \nonumber \\ {\text{(SF): } \quad \sigma _{SF}}= & {} 2(\psi _1 + \psi _{4s})\gamma + \psi _{8fs}, \nonumber \\ {\text{(SN): } \quad \sigma _{SN}}= & {} 2(\psi _1 + \psi _{4s})\gamma ,\nonumber \\ {\text{(NF): } \quad \sigma _{NF}}= & {} 2\psi _1\gamma ,\nonumber \\ {\text{(NS): } \quad \sigma _{NS}}= & {} 2\psi _1\gamma . \end{aligned}$$

For further details regarding the derivation of these equations, we refer the reader to Holzapfel and Ogden (2009). The simple shear stresses (23) reveal two assumptions built into the design of (2), namely for homogeneous simple shear deformations

24 $$\begin{aligned} {\sigma _{FS} \ge \sigma _{FN} \ge \sigma _{NF},}\nonumber \\ {\sigma _{SF} \ge \sigma _{SN} \ge \sigma _{NF}.} \end{aligned}$$

**Table 4:** Holzapfel–Ogden law parameter estimates from this and previous studies

Source	*a*	*b*	$a_f$	$b_f$	$a_s$	$b_s$	$a_{fs}$	$b_{fs}$	$I_{hom}$	$I_{fem}$
	(kPa)		(kPa)		(kPa)		(kPa)		(mN)	(mN)
Holzapfel and Ogden (2009)	0.059	8.023	18.472	16.026	2.481	11.120	0.216	11.436	36.143	36.825
Göktepe et al. (2011)	0.496	7.209	15.193	20.417	3.283	11.176	0.662	9.466	28.583	29.480
Wang et al. (2009)	0.2362	0.810	20.037	14.154	3.7245	5.1645	0.4108	11.300	33.271	34.195
Current (hom)	0.556	7.940	33.366	14.224	2.804	0.0001	0.588	8.216	**6.804**	9.653
Current (fem)	0.962	7.510	28.649	15.806	2.044	0.010	0.122	23.027	41.622	**3.899**

$I_{fem}$ indicates the value of the fit function (15) with model stresses from a finite element model ($N =8$), and $I_{hom}$ the value of the same fit function but with model stresses computed with a homogeneous deformation model. The material parameters of the last two rows originate from homogeneous and finite element model fits, respectively, in Table 2. Note that objective functional ($I-$) values for parameter sets from other studies are obtained using the orthotropic data used in this study (experimental data), and not the data used in the studies the parameter sets originate from (digitized data). The minimum fit values in the groups $I_{fem}$ and $I_{hom}$ are highlighted in bold

Out of the six datasets, only one is consistent with these orderings, namely the 6th one, which was used here under the label transversely isotropic. In this dataset the stress–strain relationship is typical of a transversely isotropic material with a stiffer fiber direction. In several other cardiac mechanics simulation studies Krishnamurthy et al. (2013), Gjerald et al. (2015), Finsberg et al. (2015), the Holzapfel and Ogden energy functional (2) has been simplified to model transversely isotropic behavior by removing the terms involving the invariants $I_{4s}, I_{8fs}$. For such a simplified model, one could use the parameter estimates for $a,b, a_f, b_f$ that we obtained from the Transversely Isotropic dataset.

However, the Holzapfel and Ogden model was originally proposed to model orthotropic behavior. This motivates also targeting a dataset displaying fully orthotropic properties. In particular, dataset 2 in Figure 1 is such and compares well with Figure 6 of Dokos et al. (2002) and Figure 2 of Holzapfel and Ogden (2009). By switching the *SF* and *SN* curves of Dataset 2, we were able to reinterpret this data in a way that is consistent with the interpretation in Holzapfel and Ogden (2009), and the shear stiffness orderings (24).

### Discussion of optimal material parameter values

We have obtained two sets of material parameters: one corresponding to an orthotropic case and one corresponding to a transversely isotropic case. We observe that for both sets of material parameters, the $b_s$ parameter essentially vanishes. For the Transversely Isotropic case, both $a_s$ and $b_s$ essentially vanish, which is in excellent agreement with the transversely isotropic stress–strain pattern. Furthermore, we note that the magnitude of both $a_s$ and $b_s$ parameters in the best fitting parameter sets presented in Table 3 are very small. In light of the shear stress calculations (23), we can see that the $a_s$ and $b_s$ parameters are related to the degree of extra stiffness in the sheet direction over the sheet normal direction. Indeed when we examine the shear data, Figure 3, we can see that the $SN-SF$ curves are only slightly stiffer than the $NF-NS$ curves, which explains why the optimal values of $a_s$ and $b_s$ are so small.

Comparing the orthotropic material parameter values to the previously published values in Table 4, we observe that the fit of our material parameters is significantly better, as expected. By using a finite element model, we have been able to relax the homogeneous shearing angle assumption and more realistically model the motion of the cubes in the shearing experiment. We note that our material parameters differ from those previously published and also that there is a significant variability in the parameter values previously reported. Some of this variability is most likely due to the differences in the selection of points during the digitization of [Figure 2 of Holzapfel and Ogden (2009)], which was done in the studies whose material parameter sets we compare in Table 4. By using original data from the shearing experiment, we were able to remove the uncertainty due to digitization in our parameter estimates. Finally we note that even after the SF-SN curves are swapped in Dataset 2 of Figure 1, there are still minor differences when compared to [Figure 7 of Holzapfel and Ogden (2009)] and [Figure 3 of Göktepe et al. (2011)] and [Figure 4 of Wang et al. (2013)]. This also explains why our parameter sets differ from those calculated in the previous studies.

### Computing functional gradients in cardiac mechanics

Figure 4 demonstrates that the computational cost of the adjoint gradient computation is comparable to that of a single iteration of the nonlinear solution algorithm of (5) for larger system sizes. For smaller system sizes, the cost of symbolic computation and the cost of residual and Jacobian assembly contribute significantly yielding higher ratios as expected. Wang et al.’s 2013 simulations of a human left ventricle in diastole use system sizes of approximately 100,000 degrees of freedom (Wang et al. 2013). Given the trend in Fig. 4, we can expect that the adjoint method and solver implemented in this work will continue to be efficient at this scale and beyond.

Comparatively, assuming the use of Newton’s method for the solution of nonlinear systems, the evaluation of a finite difference gradient requires a linear system assembly and solve for each Newton iteration, and one nonlinear solve is required per component of the gradient. Counting the 8 parameters in the Holzapfel-Ogden model (2), and assuming a typical solution of the Euler–Lagrange equation (5) takes 6 Newton iterations, we can expect the computational cost of finite difference gradient evaluation to be circa 48 times greater than that of the adjoint method.

In the optimization results of Table 2, we observed iteration counts of up to 44 for the optimization of 8 parameters using our gradient-based method. This compares favorably with the circa 7000 iterations needed to estimate 9 parameters using a global method in (Figure 5 of Wong et al. 2015).

### Implications for organ-scale image-based parameter estimation with spatially resolved material parameters

Although we have tested our adjoint-based multi-start optimization method on the 2002 shear data of Dokos et al Dokos et al. (2002), we believe our methods will provide the biggest advantage in the case of optimizing cardiac model parameters in high spatial resolution at the organ scale to MRI or echocardiographic image data. In this case the high spatial resolution would allow for detailed modeling of regional differences in tissue stiffness, which is for example present in patients with post-infarct fibrosis.

In such an application, a model parameter could be represented as a finite element function similarly to the displacement or hydrostatic pressure fields (**u**, p). Doing this would increase the number of components of the gradient $\frac{D \hat{I}}{D \mathbf{m}}$ by the number of degrees of freedom needed to spatially represent the parameter of interest. Using a finite difference or reduced order Kalman filter approach in this case would require an additional evaluation of the Euler–Lagrange equation (5) for each degree of freedom introduced, whereas the adjoint gradient formula (13) only needs to be calculated once regardless of the number of additional degrees of freedom. In the current study, the adjoint gradient is estimated to be

$$\begin{aligned} \text{(number } \text{ of } \text{ model } \text{ parameters) } \times 6 = 48 \end{aligned}$$

times faster than finite differencing. In the case of a spatially varying model parameter, the speedup is potentially a lot more significant.

When fitting material parameters to the Dokos experiment data, we were able to generate good initial guesses for the local optimization by progressively refining the mesh and using the optimal results from the previous coarser refinement level as an initial guess in the successive finer level. It would be more challenging to apply this technique using image-based ventricular geometries, due to the problem of accurately representing the geometry with few elements. As an alternative we propose the multi-start approach, which we have shown here to be accurate and viable using the Dokos experiment data.

One issue that would arise in using the multi-start approach with image-based geometries would be the choice of the number of multi-start points; using less points is more computationally efficient, while using more is potentially more robust. Possible solutions are the use of optimal stopping criteria Boender and Kan (1987) or more sophisticated local-global searches Tsai et al. (2003), Goldberg and Voessner (1999).

## Conclusions

In this work, we have presented a new application of efficient gradient-based optimization methods in the context of estimating cardiac hyperelastic material parameters from experimental data. In particular, we have demonstrated how an adjoint solution can greatly speed up the evaluation of functional gradients. These methods have produced two new sets of material parameter values that yield simulated stress–strain curves that fit closely to orthotropic and transversely isotropic shear data. For future parameter estimation, studies using image-based geometries and a local search algorithm, multi-start or a similar method should be used in order to avoid suboptimal minima.
